# Reestablishment of Social Hierarchies in Weaned Pigs after Mixing

**DOI:** 10.3390/ani10010036

**Published:** 2019-12-23

**Authors:** Xian Tong, Chunyan Shen, Ruonan Chen, Siyuan Gao, Xinpeng Liu, Allan P. Schinckel, Bo Zhou

**Affiliations:** 1 College of Animal Science and Technology, Nanjing Agricultural University, Nanjing 210095, China; 2017105081@njau.edu.cn (X.T.); 2016105082@njau.edu.cn (C.S.); 2016105033@njau.edu.cn (R.C.); 2018105082@njau.edu.cn (S.G.); 2018805122@njau.edu.cn (X.L.); 2 Department of Animal Sciences, Purdue University, West Lafayette, IN 47907-2054, USA; aschinck@purdue.edu

**Keywords:** dominance hierarchy, pig, sex, backtest, temporal dynamic

## Abstract

**Simple Summary:**

Aggression after mixing has negative effects on growth performance and animal welfare in pigs. To analyze the temporal dynamics of social hierarchy formation and maintenance, a total of 102 weaned pigs (47 females and 55 barrows) were selected and blocked by sex and body weight. The pigs were mixed into 10 single-sex pens (10 to 11 pigs/pen) avoiding original littermates. Their behavior was recorded and observed for 72 h after mixing for the analysis of dominance hierarchy indices in each pen. The three hierarchical indices: I&SI, Elo rating, and Glicko rating, were associated with each other (|*r*| = 0.681~0.942, *p* < 0.001). I&SI was associated with the logarithms of frequency of active attack (|*r*| = 0.65, *p* < 0.05) and tended to be associated to logarithms of frequency of standoff (|*r*| = 0.48, *p* < 0.1). Elo rating, and Glicko rating were associated with logarithms of duration of being bullied (|*r*| = 0.393~0.401, *p* < 0.05). In addition, Glicko rating tended to be associated with the logarithms of duration of active attack and frequency of active attack (|*r*| = 0.416~0.439, *p* < 0.1). Multiple linear regression analyses of logarithms of dyadic behavior indicators for hierarchical indices indicated the same effects. The rank of pigs became stable earlier (*p* < 0.001) in females (23.06 ± 4.15 h post mixing) than in barrows (40.55 ± 4.71 h post mixing). The first ranked pig quickly appeared within a few hours after mixing and remained stable. Our study provided new insights into the re-establishment of social hierarchies in weaned pigs after mixing.

**Abstract:**

Pigs are animals that live in groups and have social hierarchies within the group. After mixing, they can re-establish social hierarchies within several days through fighting. Dominance hierarchical indices, such as I&SI, Elo rating, and Glicko rating, have been used to analyze social hierarchies of some social animals but not pigs. I&SI index involves iterative calculations that first minimize the number of inconsistencies (I) in a dominance matrix, and then minimize the strength of those inconsistencies (SI). Elo rating and Glicko rating indices are based on the sequence in which interactions occur, and continuously update ratings by looking at interactions sequentially. To study the temporal dynamics of social hierarchy formation and maintenance in weaned pigs after mixing, a total of 102 pigs (47 females and 55 barrows) were selected by similar body weight and mixed in 10 pens (10 or 11 females or barrows per pen). Their behavior was recorded and observed for 72 h after mixing. Results showed that hierarchical indices I&SI, Elo rating, and Glicko rating were associated with each other (|*r*| = 0.681 ~ 0.942, *p* < 0.001). I&SI was associated with logarithms of frequency of active attack (|*r*| = 0.65, *p* < 0.05) and tended to associated with logarithms of frequency of standoff (|*r*| = 0.48, *p* < 0.1). Elo rating, and Glicko rating were associated with the logarithms of duration of being bullied (|*r*| = 0.393~0.401, *p* < 0.05). In addition, Glicko rating tended to be associated with the logarithms of duration of active attack and frequency of active attack (|*r*| = 0.416~0.439, *p* < 0.1). Multiple linear regression analyses of logarithms of dyadic behavior indicators for three hierarchical indices indicated the same effects. The time (hours) to achieve social stability of pigs after mixing was lower for females than barrows (23.06 ± 4.15 vs. 40.55 ± 4.71 h; *p* < 0.001). The most dominant pig (the first ranked) in each pen quickly appeared within a few hours after mixing and remained stable. Overall, our study demonstrated that the ranks calculated by the three dominance hierarchical indices: I&SI, Elo rating, and Glicko rating, were consistent and partially associated with part of the dyadic behavioral indicators in weaned pigs after mixing.

## 1. Introduction

Dominance hierarchies emerge in a group because animals have to compete for limited resources, such as food, territory, and mates [1,2]. The formation of social organization is based on the development of dominance hierarchies in a pig group [3]. In commercial pig farms, pigs are often regrouped after they are transferred between different production stages. Regrouping often starts at three to four weeks of age when piglets are weaned and moved to the weaner group where they are mixed from multiple litters [4]. Mixing is repeated when pigs are moved from the weaner groups to grower and finisher housing [5]. Mixing breaks apart the original social hierarchy, which results in post mixing aggression, a means of reestablishing a social hierarchy amongst unfamiliar pigs [6,7]. Previous studies found dominance relationships of pigs were re-established within 48 to 72 h after regrouping [6,8].

Aggression after mixing has long-term negative effects on livestock productivity and welfare [9]. For example, aggression decreases food intake and weight gain, increases injuries, and in severe cases, increases mortality [10,11]. The study of post mixing aggression is usually based on dyadic behavioral traits, which describe the direct interactions between two animals [12,13]. Dominance hierarchies are also the result of agonistic dyadic interactions between group members where there is a winner (dominant) and a loser (subordinate) [14].

**Figure 1:**
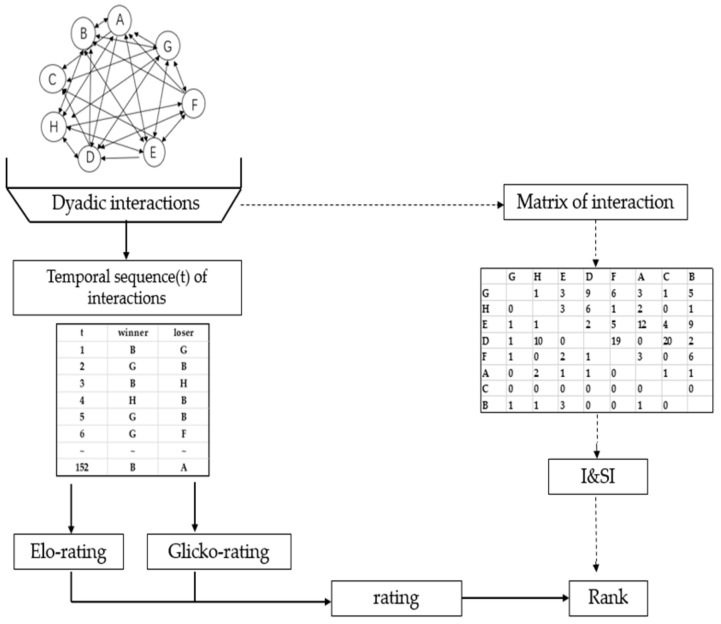
Diagram highlighting the different steps required to infer dominance hierarchies. Data shown are based on 152 interactions simulated among eight individuals from pen A. The direction of the arrows indicated the direction of the attack. The outcome of dyadic agonistic interactions between individuals was recorded either in the form of a matrix or as a temporal sequence of winners and losers.

The importance and prevalence of dominance hierarchies in nature [14] have led to the development of methods for inferring dominance hierarchies from social interactions. These methods can be classified into those estimating the rank of the individuals (I&SI [15]) and those estimating non-integer indices of success or ratings, from which individuals can further be ranked if required (Elo rating [16]; Glicko rating [17]; Figure 1). Glicko rating, an extension of the Elo dynamic paired comparison models [15], refines the Elo-rating by accounting for uncertainty in the dominance estimation.

Sociometric measures, such as Landau’s index, Kendall’s index, and directional consistency index [8], have been used to study the dominance hierarchy in pigs [3,6]. However, sociometric measures are not able to capture the dynamic nature of hierarchy formation in a large social network. In recent years, dominance hierarchical indices I &SI [15], Elo-rating [16], and Glicko rating [17] have been used in rhesus macaques (*Macaca mulatta)* [18,19], fallow deer (*Dama dama*) [20], Arctic wolves (*Canis lupus arctos*) [21], and mice [17,22]. Before the present study, these three hierarchical indices had not been used in pigs, which are also social animals [3].

Sex is one of the most common factors that influence animal behavior [18,23]. In mice, sex affected the formation of social hierarchies [23]. Sex also influenced aggressive behavior in pigs [24]. Barrows not only initiated more aggression than females [25], but also won more fights [26]. However, the effect of sex on dominance hierarchy formation is not clear in pigs.

As a behavioral test for coping style, the backtest [27] has been used to identify pigs with more aggressive behavior. A previous study found that proactive responders for the backtest tended to be more rigid in their behavior, whereas passive responders seemed to more flexible [28,29]. No research has been conducted which has evaluated the extent to which the backtest response in pigs is associated with their dominance hierarchy.

We hypothesized that (i) the dominance hierarchies determined by I&SI, Elo rating, and Glicko rating indices were linked, (ii) these three hierarchical indices were associated with dyadic behavior traits, such as duration and frequency of active attack, being bullied, and standoff, (iii) the score of backtest could be used to predict dominance hierarchy in pigs, and (iv) the formation of dominance hierarchies would be different between gilts and barrows. The objectives of the present study were to explore the temporal dynamics of dominance hierarchy formation and maintenance in weaned pigs.

## 2. Materials and Methods

All procedures related to the management and care of animals in this experiment were approved by the Nanjing Agricultural University Animal Care and Use Committee (SYXK Su 2017-0007).

### 2.1. Animals and Housing

This experiment was conducted at Huaiyin pig breeding farm (Huaian, Jiangsu, China). All pigs were processed using standard practices during the lactation period, including teeth clipping, tail docking, and ear notching at 3 days of age, and the males were castrated at 7 days of age. Piglets were weaned at 35 days of age and moved to empty pens with their original pen-mates in a nursery room at 2 days before mixing. First, weaned pigs were blocked by sex and body weight. A total of 102 pigs (47 females and 55 barrows) from 37 litters were selected by similar body weight and mixed in 10 pens (10 or 11 females or barrows per pen) of dimension 2.5 × 2.2 m. All pens were equipped with slatted floors, stainless-steel vibratory feeders and nipple drinkers to allow ad libitum access to feed and water. All tests were conducted in the same room with natural light and dark cycle (12 h:12 h). The same animal caretaker performed all tests. At 72 h after mixing, all pigs were weighed again to calculate the average daily gain (ADG).

### 2.2. Backtest

At 35 days of age, all experimental piglets were subjected to the backtest twice in a separated room in the nursery barn before mixing. Piglets were placed in a supine position for one minute to observe their behavior response, and the frequency of struggling attempts was recorded [30].

### 2.3. Behavioral Observations

Pig behavior was recorded continuously for 72 h post mixing using a digital video recording system (Hikvision DS-2CE56C2P-IT3 3.6 mm; Hikvision network hard disk video recorder DS-7808HW-E1/M; Hikvision Digital Technology Co. Ltd., Hangzhou, China). Video cameras were installed over each pen, permitting a bird’s eye view of the whole pen. Pigs were individually spray marked (7CF, Shenzhen Zhaoxin Energy Co., Ltd., Shenzhen, China) on the back and both sides of the body before mixing. The definition of dyadic behavioral traits and the distinction between given and received behaviors are shown in Table 1. A fight had to last for 3 s. Two fights between the same pair of pigs had to be at least 8 s. Otherwise, they were treated as a single fight event [31]. The recording speed and playback speed was 30 frames/s. All video images were observed by two trained technicians. A part of behavior data was randomly selected and verified by another trained technician to ensure the correctness of the behavioral data observation.

### 2.4. Statistical Analysis

I&SI values were calculated using R package ‘compete’ v.0.1 [35]. The accumulated frequencies of wins and losses of pigs were aggregated into a separate win/loss frequency sociomatrix, where the winners were listed in rows and losers in columns for each pen. A binarized 1/0 (win/loss) sociomatrix was derived from the frequency of the win/loss matrix. According to the method of a previous study [36], in each cell of the matrix, the frequency of win/loss was assigned “1” to the pigs in rows who won more often against pigs in columns, and losers in columns for each pen. If the fight was tied, both pigs received “0”. Using the binary win/loss sociomatrices, the rank orders of individuals in each pen were calculated [15] (Supplementary Materials Figure S2).

Elo rating values were calculated using R package ‘Elo rating’ [37], which reflects an individual’s success in agonistic interactions and is based on temporal sequences of decided (clear winner and loser) agonistic interactions [16]. At the beginning of the observation period, each individual in a group starts with a rating of 1000, which is updated, i.e., increased or decreased, after each agonistic interaction based on the outcome of the interaction (won or lost).

Higher-rated individual wins:

Winner Rating _new_ = Winner Rating _old_ + (1 − p) × k

Loser Rating _new_ = Loser Rating _old_ − (1 – p) × k

Lower-rated individual wins (against the expectation):

Winner Rating _new_ = Winner Rating _old_ + p × k

Loser Rating _new_ = Loser Rating _old_ − p × k

where p is the expectation of winning for the higher-rated individual, which is a function of the absolute difference in the ratings of the two interaction partners before the interaction [38] and k is a constant and determines the number of rating points that an individual gains or loses after a single encounter. The present ratings of both opponents and a determined factor, k = 100, followed a previous study [16].

Glicko ratings were calculated using ‘Player Ratings’ package v.1.0 in R [39]. The temporal changes in individual dominance ratings of each piglet in each pen were calculated using Glicko ratings index [22,40]. Glicko ratings are an extension of the Elo dynamic paired comparison models [16], whereby a cardinal dominance score for each individual is derived based on the temporal sequence of wins and losses. Briefly, all individuals begin with the same initial rating (2200) and rating deviation (300). Rating points increase or decrease for each individual determined by a function accounting for the ratings difference between opponents as well as the measure of certainty of each opponent’s rating (deviation of their ratings) [40].

Hierarchical indices I&SI, Elo rating, and Glicko ratings of pigs were calculated using R v.3.2.2 [41]. Since data of dyadic behavior traits, such as the duration and frequency of active attack, being bullied, and standoff, were non-normally distributed, logarithms of these data were used in the analyses. Partial correlations between hierarchical indices and dyadic behavior indicators were analyzed with other dyadic behavior indicators as the covariates. Multiple linear regression analyses of dyadic behavior indicators on hierarchical indices were analyzed using SPSS 25.0 (SPSS Inc., Chicago, IL, USA). The data of time (hours) to social stability of each pig after mixing were analyzed using PROC GLIMMIX procedure in SAS 9.4 (SAS Institute Inc., Cary, NC, USA). The fixed effects were sex, parity, boar, initial body weight and interaction between initial body weight and sex, and pen as a random effect. Spearman rank correlations between Glicko ratings of each hour and Glicko ratings of the last hour (72 h post mixing) were analyzed using SPSS 25.0 (SPSS Inc., Chicago, IL, USA). The results are presented as mean ± SEM and *p* < 0.05 was considered significant and 0.05 < *p* < 0.10 was considered a tendency.

## 3. Results

### 3.1. Similarity between I&SI, Elo Rating Indices, and Glicko Ratings

**Figure 2:**
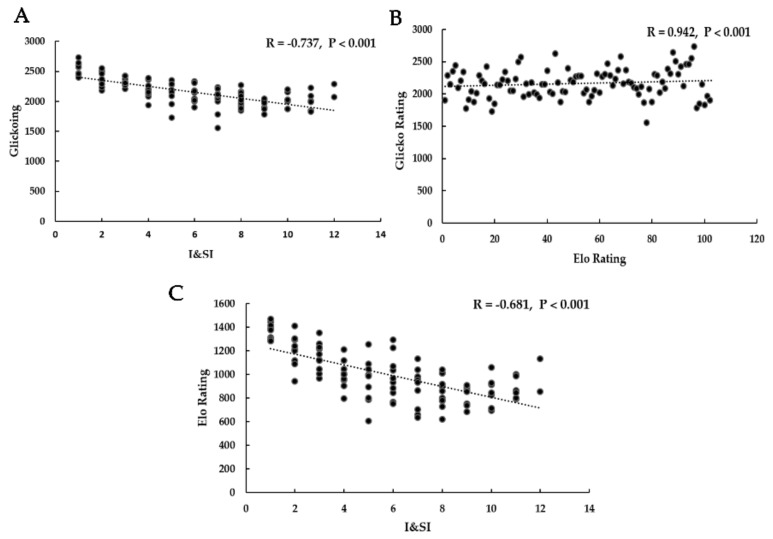
Spearman rank correlations between I&SI, Elo rating, and Glicko rating indices. (**A**) The linear relationship between I&SI and Glicko rating; (**B**) The linear relationship between Elo rating and Glicko rating; (**C**) The linear relationship between I&SI and Elo rating.

The dominance hierarchies of all experimental pigs were calculated by I&SI, Elo rating, and Glicko ratings indices. The win/loss frequency sociomatrices and binarized sociomatrices are shown in Supplementary Materials Figures S1 and S2, respectively. Significant correlations were found between I&SI, Elo rating, and Glicko rating indices (Figure 2, |*r*| _I&SI and Glicko rating_ = 0.737, *p* < 0.001; |*r*| _Elo rating and Glicko rating_ = 0.942, *p* < 0.001; |*r*| _I&SI and Elo rating_ = 0.681, *p* < 0.001). 

### 3.2. Partial Correlation Analyses of Hierarchical Indices with Dyadic Behavioral Indicators, Backtest Score, Body Weight, and Average Daily Gain (ADG)

**Figure 3:**
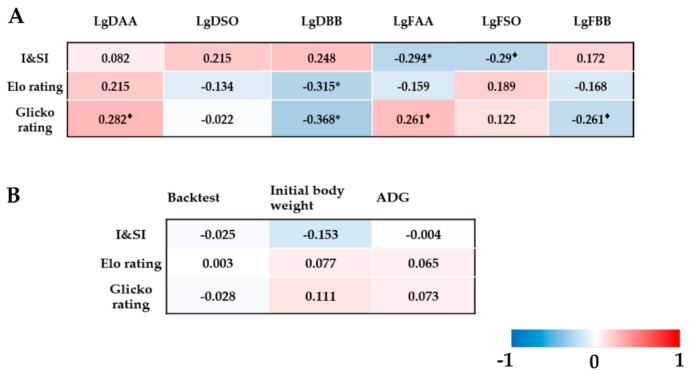
(**A**) Partial correlation analyses between hierarchical indices (I&SI, Elo rating, or Glicko rating) and logarithms of dyadic behavioral indicators. (**B**) Partial correlation analyses between hierarchical indices (I&SI, Elo rating, or Glicko ratings) and backtest score, initial body weight or average daily gain (ADG). “*” represents *p* < 0.05. “^♦^” represents 0.05 < *p* < 0.1. Blue color represents negative correlation and red color represents positive correlation. Cells were colored on a gradient from blue (r = −1) to red (r = 1), where the colors were related to the correlation coefficients. DAA = Duration of active attack; DSO = Duration of standoff; DBB = Duration of being bullied; FAA = Frequency of active attack; FSO = Frequency of standoff; FBB = Frequency of being bullied.

As shown in Figure 3A, I&SI was significantly associated with the frequency of active attack (|*r*| = 0.65, *p* < 0.05) and tended to be associated with the frequency of standoff (|*r*| = 0.48, *p* < 0.1). Elo rating and Glicko rating were associated with the duration of being bullied (|*r*| = 0.393~0.401, *p* < 0.05). In addition, Glicko rating tended to be associated with the duration of active attack and frequency of active attack (|*r*| = 0.416~0.439, *p* < 0.1). No relationships were found between hierarchical indices and duration of being bullied, backtest score, initial body weight or ADG (*p* > 0.1) (Figure 3B).

### 3.3. Multiple Linear Regression Analyses of Logarithms of Dyadic Behavior Indicators for Hierarchical Indices

As shown in Table 2, logarithms of frequency of active attack (*p* = 0.050) and standoff (*p* = 0.053) tended to influence hierarchical index ISI. The logarithm of duration of being bullied (*p* = 0.035) influenced Elo rating, the logarithm of duration of being bullied (*p* = 0.013) influenced Glicko rating. Meanwhile, the logarithms of duration (*p* = 0.060) and frequency of active attack (*p* = 0.083) and being bullied (*p* = 0.083) tended to influence Glicko rating. These results are all consistent with the partial correlation analyses.

### 3.4. Temporal Dynamics of Individual Glicko Ratings

**Figure 4:**
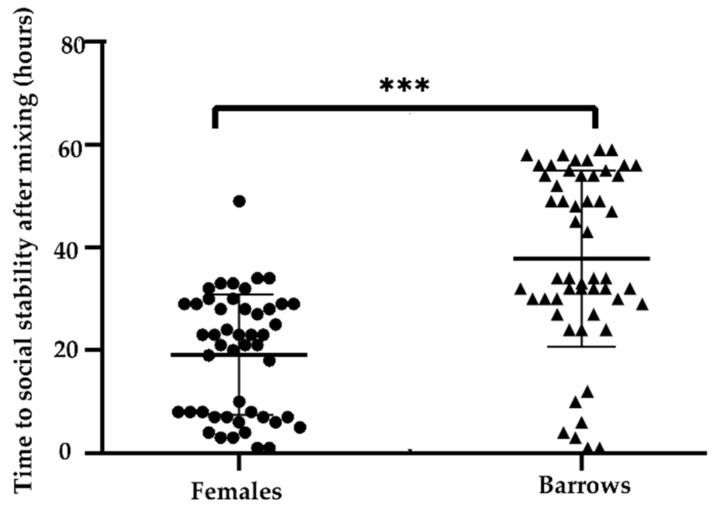
The difference of time to social stability after mixing between females and barrows. “***” represents *p* < 0.001.

**Figure 5:**
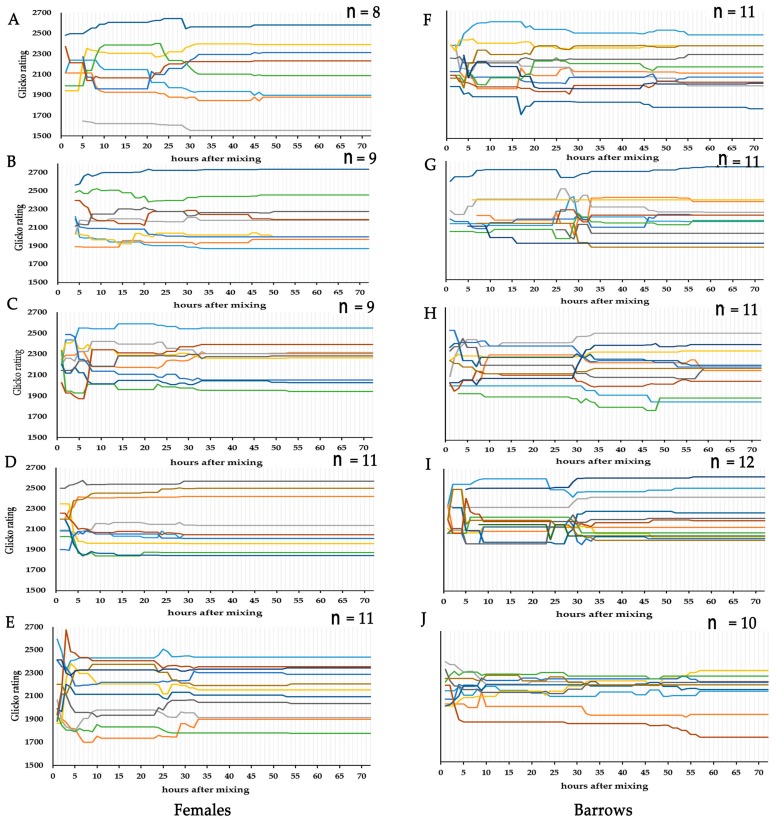
Temporal dynamics of individual Glicko ratings by pen. The change in individual Glicko ratings during 72 h post mixing for all pens (**A**–**J**). Each line represents the ratings of one individual with different colors representing individual IDs in each pen. Ratings were recalculated for individuals according to their agonistic interactions and were plotted on the y-axis against hours after mixing on the x-axis. Vertical dashed lines represent the end of each hour of observations.

As shown in Figure 4, the time (hours) to achieve social stability of pigs after mixing was less for females than barrows (23.06 ± 4.15 vs. 40.55 ± 4.71 h; *p* < 0.001). The first ranked individual at the end of the observation time had already become the first rank individual after 10 h post mixing in all female pens and three barrow pens (pen F, G, and H). The first ranked individual in barrow pen (I) did not change after 24 h post mixing. In the barrow pen (J), the eventual dominant individual achieved the first rank 55 h post mixing (Figure 5).

**Figure 6:**
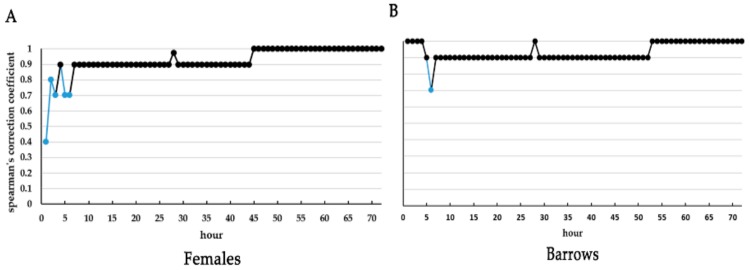
Correlation coefficients between Glicko ratings at each hour and Glicko rating at 72 h post mixing for the first ranked females (**A**) and barrows (**B**). Blue colors indicate there is no correlation of Glicko ratings between the present hour and 72 h post mixing (*p* > 0.05); Black colors indicate there is a significant correlation of Glicko ratings between the present hour and 72 h post mixing (*p <* 0.05). Y-axis representing the Spearman’s ranks correlation coefficient of Glicko ratings between the present hour and 72 h post mixing.

For the first ranked pigs, Glicko ratings of females calculated at each hour after hour 7 were positively correlated with that at 72 h post mixing (*p* < 0.05). Meanwhile, all Glicko ratings of barrows calculated at each hour, except hour 4 post mixing, were positively related (*p* < 0.05) to that at 72 h post mixing (Figure 6). This indicates that the first ranked pig in each pen quickly appeared and remained stable within a few hours after mixing.

## 4. Discussion

### 4.1. Similarity between I&SI, Elo Rating, and Glicko Rating Indices

As common approaches, dominance hierarchical indices [42] I&SI, Elo rating, and Glicko rating have been used to analyze the hierarchies of macaques [19] and mice [16,17]. In the present study, we first used I&SI, Elo rating, and Glicko rating indices to analyze the dominance hierarchies of weaned pigs after mixing. In our present study, I&SI, Elo rating, and Glicko rating indices were related to each other, suggesting that all these three dominance hierarchy indices are consistent and can be used to analyze the hierarchies of pigs in a group.

### 4.2. Correlation Analyses of Dominance Hierarchical Indices with Behavioral Indicators, Backtest Score, and Body Weight

In this study, the three dominance hierarchical indices: I&SI, Elo rating, and Glicko rating, were positively associated with aggressive behavior in weaned pigs post mixing, which is in agreement with a previous study [31]. Previous studies have shown that backtest score was not consistently associated with aggression in piglets [27,28,30]. In our present study, backtest scores were not related to any hierarchical indices, suggesting that the scores of backtest could not be used to predict the hierarchies of weaned pigs after mixing.

In the present study, the initial body weight and ADG of weaned pigs during the 72 h post mixing were not associated with any hierarchical indices, which is in agreement with a previous study [43]. However, ADG might be influenced by the short experimental period in our present study.

### 4.3. Temporal Dynamics of Individual Glicko Ratings

Glicko rating index has been used to analyze the temporal changes in the formation and maintenance of dominance hierarchies [1]. In the present study, dominance relationships were reestablished within 48 to 72 h after regrouping, which is in agreement with a previous study [8].

The time (hours) to achieve social stability of pigs after mixing was lower for females than barrows in the present study. A previous study found that barrows had a longer attack latency than that of gilts [44], which suggests that sex influences the formation of dominance hierarchies in pigs.

In the present study, Glicko ratings predicted their final Glicko ratings for the first ranked pig in each pen after 7 h post mixing. The first ranked individuals quickly appeared and remained stable within a few hours after mixing, which is consistent with a previous study [6].

Our present study provided a new insight into aggressive behavior of pigs after mixing. We found relatively precise time points when dominance hierarchies become stable in females and barrows after mixing. However, their age [45], breed [46], pen space [47], and feed could also impact the time to stabilize hierarchies. Further studies should evaluate other factors influencing the hierarchy formation. Furthermore, consideration of comparable and standardized social hierarchy indices which uncover processes of social hierarchy formation in pig production could improve animal welfare and productivity.

## 5. Conclusions

Consistent with our hypotheses, the three hierarchical indices: I&SI, Elo rating, and Glicko rating, were significantly associated with each other and can be used in pigs. Dominance hierarchical indices I&SI, Elo rating, and Glicko rating were partially associated with part of the dyadic behavioral indicators in weaned pigs after mixing. Contrary to our predictions, the backtest scores were not associated with hierarchical indices in weaned pigs post mixing. The time required to achieve social stability in groups of pigs after mixing was lower for females than barrows. Furthermore, the first ranked pig in each pen quickly appeared and remained stable within a few hours after mixing.

## Figures and Tables

**Table 1:** Description of dyadic behavioral traits.

Trait	Description
Active attack	In a fight, a pig demonstrates biting, pushing, chasing [32].
Being bullied	When the recipient pig suffers from biting and head-knocking performed by the actor pig and the recipient moved away without retaliation, it was identified as a being bullied [33].
Standoff	Two pigs stand side by side, shoulder by shoulder, and one pig throws its head to the head or neck of the other pig. Not including active attack and being bullied.
Win/lose/draw	A pig showed a submissive manner, such as stopping fighting, turning away from an attack, trying to flee or was displaced from the location, was defined as a loser, the other pig in the fight was defined as a winner [34]. If there was no clear outcome, the fight was designated as a draw [31].

**Table 2:** Regression coefficients and standard errors for multiple linear regression analyses of logarithms of dyadic behavior indicators for hierarchical indices ISI, Elo rating, and Glicko rating.

Covariates	Unstandardized Coefficients B	Unstandardized Coefficients Standard Error	Test Statistic (*t* Value)	Significance Level (*p*-Value)
Dependent Variable: ISI				
LgDAA	0.682	1.269	0.537	0.594
LgDSO	1.611	1.113	1.447	0.155
LgDBB	2.324	1.387	1.676	0.101
LgFAA	−4.644	2.302	−2.017	0.050 ^♦^
LgFSO	−3.634	1.828	−1.989	0.053 ^♦^
LgFBB	2.623	2.297	1.142	0.260
Dependent Variable: Elo rating				
LgDAA	109.617	75.800	1.446	0.155
LgDSO	−58.860	66.475	−0.885	0.381
LgDBB	−180.248	82.824	−2.176	0.035 *
LgFAA	145.088	137.488	1.055	0.297
LgFSO	137.923	109.138	1.264	0.213
LgFBB	−152.971	137.182	−1.115	0.271
Dependent Variable: Glicko rating				
LgDAA	130.327	67.489	1.931	0.060 ^♦^
LgDSO	−8.696	59.186	−0.147	0.884
LgDBB	−191.441	73.742	−2.596	0.013 *
LgFAA	217.211	122.413	1.774	0.083 ^♦^
LgFSO	78.576	97.171	0.809	0.423
LgFBB	−216.628	122.140	−1.774	0.083 ^♦^

“*” represents *p* < 0.05. “^♦^” represents 0.05 < *p* < 0.1. DAA = Duration of active attack; DSO = Duration of standoff; DBB = Duration of being bullied; FAA = Frequency of active attack; FSO = Frequency of standoff; FBB = Frequency of being bullied.

## Supplementary Materials

The following are available online at https://www.mdpi.com/2076-2615/10/1/36/s1, Figure S1: Frequency Win-Loss Sociomatrices. Figure S2: Binarized Win-Loss Sociomatrices.
