# Supplementary material for: Reestablishment of Social Hierarchies in Weaned Pigs after Mixing
Source: Animals (Basel). 2019 Dec 23;10(1):36. doi: 10.3390/ani10010036 (PMC7022989; doi:10.3390/ani10010036)
Supplement: Supplementary file 1 [file animals-10-00036-s001.pdf]

animals

Supplementary Materials: Reestablishment of Social Hierarchies in Weaned Pigs after Mixing

Xian Tong [1], Chunyan Shen [1], Ruonan Chen [1], Siyuan Gao [1], Xinpeng Liu [1], Allan P. Schinckel [2] and Bo Zhou [1,*]

[1] College of Animal Science and Technology, Nanjing Agricultural University, Nanjing 210095, China; 2017105081@njau.edu.cn (X.T.); 2016105082@njau.edu.cn (C.S.); 2016105033@njau.edu.cn (R.C.); 2018105082@njau.edu.cn (S.G.); 2018805122@njau.edu.cn (X.L.)

[2] Department of Animal Sciences, Purdue University, West Lafayette, IN 47907-2054, USA; aschinck@purdue.edu

* Corresponding: zhoubo@njau.edu.cn

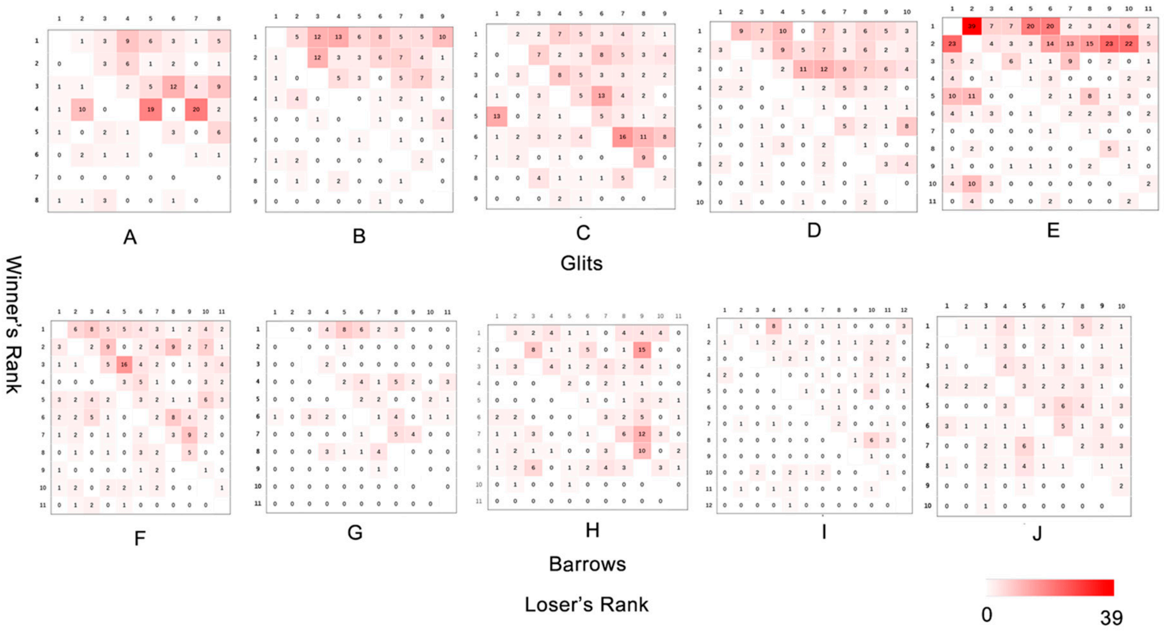

Figure S1. Frequency Win-Loss Sociomatrices. Total frequency of agonistic interactions that occurred between all pairs of individuals across all pens (**A-J**) over the entire observation period. Winners of each pens are listed in rows and losers are listed in columns. Ranks were calculated using the I&SI method. Cells of each matrix are colored on a gradient from white (lowest value in total matrix) to red (highest value in total matrix).

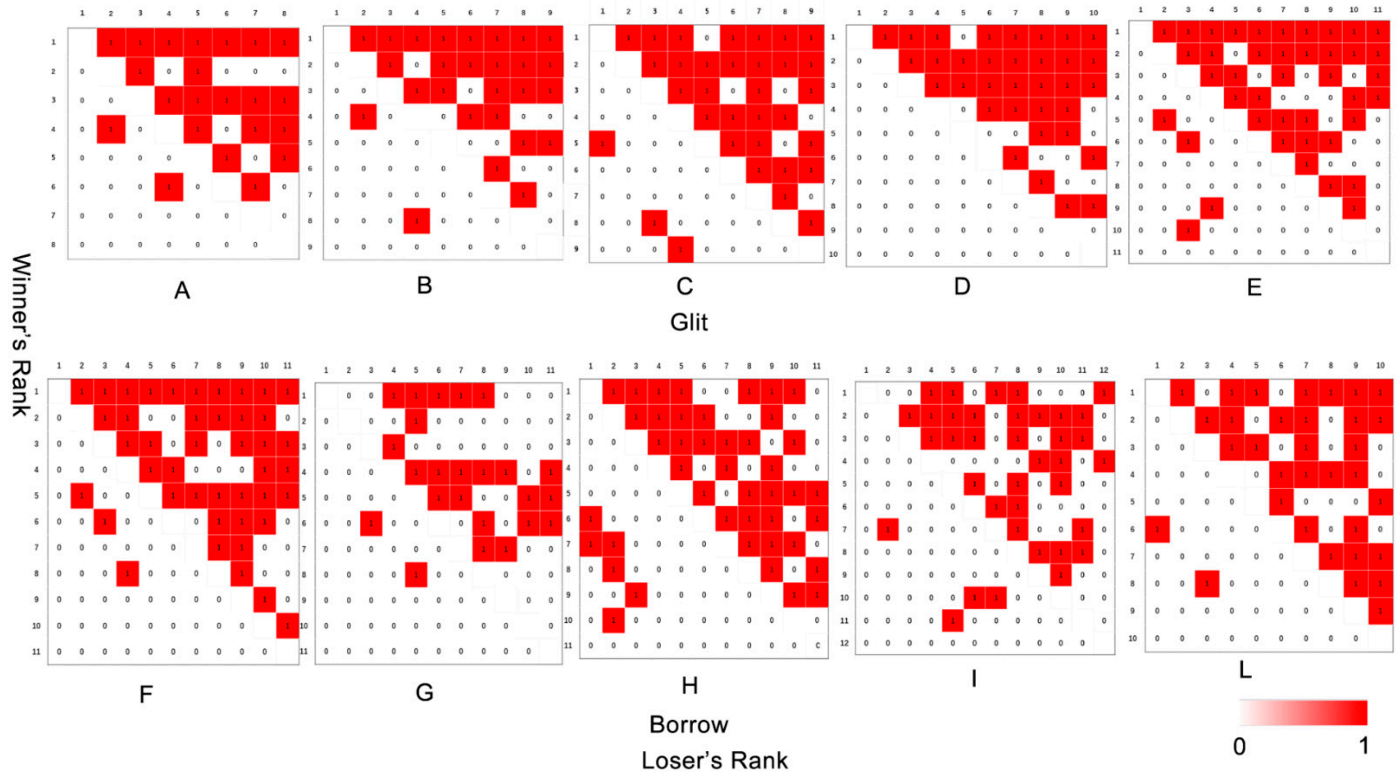

Figure S2. Binarized Win-Loss Sociomatrices. For each relationship within each pen, the winner and loser were calculated by determining which individual had the most wins over all observations. Winners of each pen (**A-J**) are listed in rows and assigned a value of 1. Losers are listed in columns. Ranks were calculated using the I&SI method.
